# Development of a biomechanical energy harvester

**DOI:** 10.1186/1743-0003-6-22

**Published:** 2009-06-23

**Authors:** Qingguo Li, Veronica Naing, J Maxwell Donelan

**Affiliations:** 1 Department of Mechanical and Materials Engineering, Queen's University, Kinston, ON, Canada, K7L 3N6; 2 Department of Biomedical Physiology and Kinesiology, Simon Fraser University, Burnaby, BC, Canada, V5A 1S6

## Abstract

**Background:**

Biomechanical energy harvesting–generating electricity from people during daily activities–is a promising alternative to batteries for powering increasingly sophisticated portable devices. We recently developed a wearable knee-mounted energy harvesting device that generated electricity during human walking. In this methods-focused paper, we explain the physiological principles that guided our design process and present a detailed description of our device design with an emphasis on new analyses.

**Methods:**

Effectively harvesting energy from walking requires a small lightweight device that efficiently converts intermittent, bi-directional, low speed and high torque mechanical power to electricity, and selectively engages power generation to assist muscles in performing negative mechanical work. To achieve this, our device used a one-way clutch to transmit only knee extension motions, a spur gear transmission to amplify the angular speed, a brushless DC rotary magnetic generator to convert the mechanical power into electrical power, a control system to determine when to open and close the power generation circuit based on measurements of knee angle, and a customized orthopaedic knee brace to distribute the device reaction torque over a large leg surface area.

**Results:**

The device selectively engaged power generation towards the end of swing extension, assisting knee flexor muscles by producing substantial flexion torque (6.4 Nm), and efficiently converted the input mechanical power into electricity (54.6%). Consequently, six subjects walking at 1.5 m/s generated 4.8 ± 0.8 W of electrical power with only a 5.0 ± 21 W increase in metabolic cost.

**Conclusion:**

Biomechanical energy harvesting is capable of generating substantial amounts of electrical power from walking with little additional user effort making future versions of this technology particularly promising for charging portable medical devices.

## Introduction

From mobile phones to laptop computers, society has become increasingly dependent on portable electronic devices [1]. Because batteries almost exclusively power these devices, and the energy per unit mass in batteries is limited, there is a trade-off between device power consumption, battery weight and duration of operation. For example, while a typical mobile phone consumes a modest 0.9 W electrical requiring a 18 g Li-ion battery for 3 hours of talk time [2], a typical laptop computer requires a 720 g Li-ion battery to satisfy its 28 W electrical power needs, lasting less than 4 hours [3]. This trade-off is particularly severe in the design of powered prosthetic joints that need to be lightweight while performing their sophisticated task over a full day of typical use. The manufacturers of the C-leg, Rheo Knee and Proprio Foot indicate that their devices operate for more than 36 hours from a single charge of a battery that weighs about 230 g battery equating to an average power consumption of less than 1 W electrical [4-6]. Substantial improvement to the operating time or performance of a portable device, while avoiding the unattractive solution of simply heavier batteries, requires an alternative to current battery technology [1].

Human power is an attractive energy source. Muscle converts food into positive mechanical work with peak efficiency of approximately 25%, comparable to that of internal combustion engines [7]. The work can be performed at a high rate, with 100 W mechanical easily sustainable by an average person [8]. Food, the original source of the metabolic energy required by muscles, is nearly as rich an energy source as gasoline and approximately 100 fold greater than batteries of the same weight [9]. Given these attractive properties, it is not surprising that a number of inventions have focused on converting human mechanical power into electrical power. These include hand crank and bicycle generators as well as windup flashlights, radios, and cell phone chargers [10]. One major drawback of these devices is that they require dedicated power generation by the user, limiting the time available to produce power and thus the amount of useful energy that can be generated.

In contrast, biomechanical energy harvesters generate electricity from people as they go about their activities of daily living resulting in power generation over much longer durations [1]. Self winding watches, for example, use arm motion to excite a load which drives a generator producing approximately 5 μW electrical [10,11]. Employing the same basic principle as the self winding watch, Rome et al.'s energy harvesting backpack uses the mass of the moving pack to drive a rotary-magnetic generator [12,13]. This impressive device produced 7.4 W electrical from a 38 kg load during fast walking and approximately 0.5 W electrical at more modest loads and speeds. Harvesting substantial energy from an external load requires that a relatively heavy external mass be excited to relatively fast speeds–an energetically costly scenario that only makes sense if one is already obligated to carry the load. A promising alternative is to use the body's own mass to generate electricity. The most popular embodiment of this principle harvests energy from the compression of the shoe sole as the leading leg accepts the weight of the body during walking. The most successful design uses a dielectric electroactive polymer to generate 800 mW electrical [14].

We recently developed a biomechanical energy harvester for generating electricity during human walking [15]. Our device differed from previous devices in two main ways. First, the device took advantage of the fact that much of the displacement during walking occurs at body joints and harvested energy from knee motion rather than from an external load or the compression of the shoe sole. Second, the device selectively engaged power generation to assist the body in performing negative work. Its development required an understanding of the physiology of walking and a novel design to best take advantage of the underlying physiological principles. As muscle is ultimately the origin of all energy available for biomechanical energy harvesting, the first purpose of this methods-focused paper is to explain the physiological principles that guided our design process. The second purpose is to present a detailed description of our device design with a focus on new analyses that provide further insight into its function.

## Methods

### Walking mechanics and energetics

On average, there is no net mechanical work performed on the body during walking at a constant speed on level ground as there is no net change in kinetic or potential energy. This is accomplished by a number of sources–including muscle, tendon, clothing and air resistance–contributing to perform equal amounts of positive and negative mechanical work [16]. Selectively engaging a generator at the right times and in the right locations could assist with performing negative mechanical work on the body, replacing that normally provided by other sources such as muscle. This is similar to how regenerative braking generates power while decelerating a hybrid car [17]. We have termed this form of energy harvesting *generative braking *as the electricity is not reused to directly power walking but is available for other uses [15].

In principle, generative braking can produce electricity while reducing the metabolic cost of walking. When performing positive mechanical work, active muscle fibres shorten while developing force, converting chemical energy (i.e. metabolic energy) into mechanical energy. The peak efficiency of positive muscle work is approximately 25% [7]. That is, a muscle producing 1 W mechanical requires 4 W metabolic and dissipates 3 W as heat. When performing negative work, muscle fibres develop force but are compelled to lengthen by an external force. This braking system is not passive–muscles require metabolic energy to perform negative work. The peak efficiency of negative work production is approximately -120% [7]. That is, a muscle producing -1 W mechanical requires 0.83 W metabolic and dissipates 1.83 W heat. We refer to generating electricity by increasing positive muscle work–as is the case with hand cranks and bicycle generators–as *conventional generation*. Generating electricity in this manner will cause a relatively large increase in effort, while electricity generation that results in a decrease in negative muscle work will result in a relatively small decrease in effort.

While muscles are the only source of positive work in walking, there are other sources of negative work in addition to muscle. These include air resistance, damping within the shoe sole and movement of soft tissue. These are considered passive sources of negative work in that, unlike muscle, they don't require metabolic energy to dissipate mechanical energy. While the contribution of air resistance and shoe sole damping are thought to be small during walking [18,19], the quantitative contribution of soft tissue movement to negative work is not yet clear [20]. While muscles do not perform all of the required negative mechanical work during walking, it is believed that they perform a substantial fraction [21-23]. Nevertheless, it is possible for negative work by an energy harvesting device to replace negative work by a passive source, such as soft tissue, resulting in no change in metabolic cost to the user.

**Figure 1:**
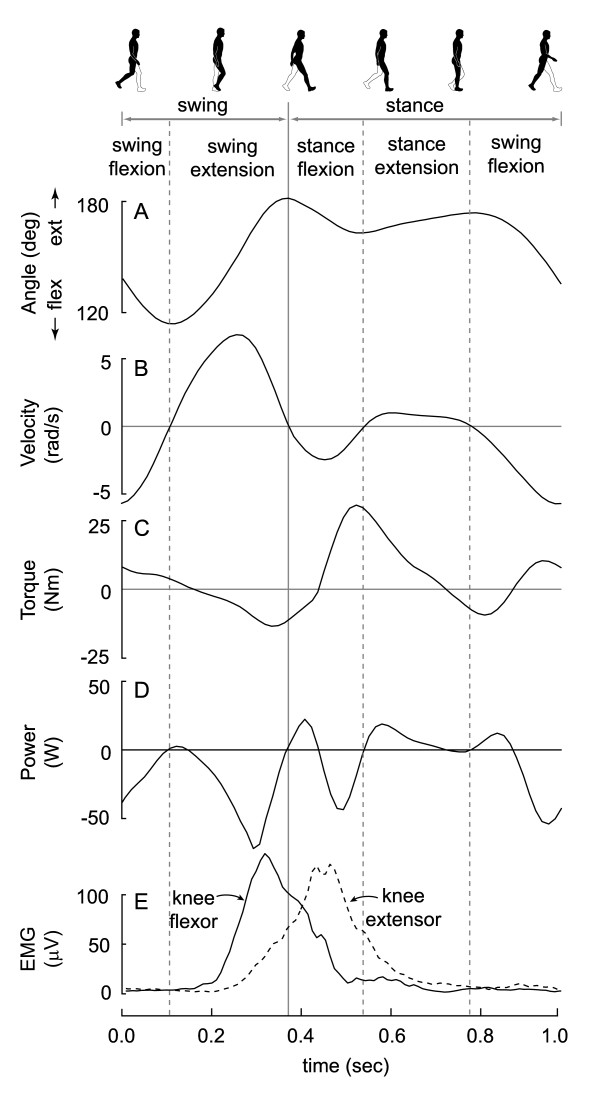
**Typical knee joint mechanics and muscle activity during walking (subject mass = 58 kg; speed = 1.3 m/s; step frequency = 1.8 Hz**. Data from [24,25]). A) Knee joint angle where 180 degrees is full knee extension. B) Knee joint angular velocity using the convention that positive angular velocity is motion in the extension direction. C) Knee joint torque with the convention that extensor muscle torques are positive. D) Knee joint power. E) Rectified and filtered electromyograms (EMG) from one knee flexor muscle (solid line) and one knee extensor muscle (dashed line).

Muscles do not act on the environment directly. Instead, muscles act on the body's skeleton which functions as a system of levers to transmit the muscle work to the rest of the body. As a consequence, rates of performing positive and negative muscle work are measured externally as positive and negative joint power [24]. Figure 1 presents knee joint power data for a single subject walking at a comfortable speed [24,25]. Mechanical power outputs at other joints can demonstrate very different patterns and power generation at all joints depends on many parameters including walking speed and the mass of the subject [24,26]. Regardless of joint or condition, joint power is typically intermittent, bi-directional, and time-varying. These characteristics represent a significant challenge for energy harvesting around joints.

It is difficult, however, to interpret muscle function from joint power alone [27]. This is for three main reasons. First, all joints are spanned by muscles that generate forces to oppose each other and these muscles can be simultaneously active. Thus, net positive joint power can result from positive and negative power production by opposing muscles. Using a generator to resist the motion of a joint may usefully assist the negative power producing muscles, even in the presence of net positive joint power. Second, muscles often cross multiple joints. An isometric muscle, or even one that is generating net positive power, may contribute to negative joint power at one joint while it simultaneously generates positive joint power at other joints [27]. Resisting the motion of a negative power producing joint may ultimately increase the positive mechanical power required of the muscles that span that joint. Third, tendons and other connective tissue can store and return elastic energy [28]. Negative joint power may be due to this elastic tissue storing mechanical power for later use. Using a generator to resist joint motion may interfere with this storage and ultimately increase the positive work required of muscle. As a consequence of the complicated physiology, claims regarding the appropriate joint and timing for exploiting generative braking are best viewed as predictions until tested experimentally.

The knee primarily performs negative work during walking making it a good candidate for generative braking. Figure 1 illustrates four main phases of knee kinematics, each delineated by a change in direction of motion: stance flexion, stance extension, swing flexion and swing extension. Beginning shortly after foot contact, the muscles that act to extend the knee are active (E) producing an extensor moment (C) during stance flexion. However, the knee is flexing (B) as the leg accepts the body weight, resulting in negative joint power (D). During stance extension, the knee extensor muscles are still generating an extensor torque and have redirected the joint motion resulting in a period of positive joint power. It is important to note that there is a delay between the measured muscle activity and the corresponding muscle force resulting in activity that precedes force generation and force generation that continues after activity ends [29]. The knee flexes towards the end of stance and continues flexing into the swing phase. For convenience, we refer to this period as swing flexion while recognizing that it begins during stance. There is primarily negative joint power production during this swing flexion due to the dominant knee extensor moment. The fourth region, and the most important one for our current purpose, is swing extension. Knee joint power is primarily negative due to the flexor moment produced by the knee flexors to slow down the extending knee prior to foot contact.

To harvest energy using generative braking, we selectively engaged power generation during swing extension. The physiological reasons for targeting swing extension are threefold. First, a large amount of negative joint work is performed during this phase. At a comfortable walking speed, for example, each leg performs approximately -8.4 J in swing extension compared to -6.3 J during stance flexion (Figure 1) [30]. Second, the swing phase negative work does not depend strongly on speed when compared to other phases. For example, swing extension work decreases by only 19% between 1.5 m/s and 1.0 m/s while stance flexion work decreases by 56% [30]. The third reason is that the negative joint power during swing extension is likely due to actual negative muscle work rather than net positive work by muscles that cross more than one joint or the storage of useful elastic energy. This is because while the knee is extending, the hip first flexes and then remains at a nearly constant angle, forcibly lengthening the active knee flexor muscles that also act to extend the hip [31]. While some of the swing extension negative work may be due elastic tissue like tendons, it is unlikely that this is returned in a useful manner because it is followed by a negative work flexion phase.

### Device Design

The biomechanics of walking presented four main challenges for designing a device to harvest energy from the motion of the knee joint. The first challenge was to determine an effective mechanism for converting biomechanical power into electrical power. This generator had to be worn on the body so it needed to be small and lightweight. The second challenge was to design a mechanism for converting the intermittent, bi-directional and time-varying knee joint power into a form suitable for efficient electrical power generation. The third challenge was to optimize the system parameters in order to maximize the electrical power generation without adversely affecting the walking motion. At any given point in the walking cycle, there is only a certain amount of knee mechanical power available–attempting to harvest too much power will cause the user to limp or stop walking while harvesting too little results in less electrical power generated. The final design challenge was to determine a mechanism for selectively engaging power generation during swing extension to achieve generative braking.

We evaluated piezoelectric, electroactive polymer, and electromagnetic generators to determine their suitability for efficient and lightweight biomechanical energy conversion. While piezoelectric material has a versatile form factor, it suffers from an inherently low mechanical to electrical conversion efficiency (often less than 5%) [32]. In addition, input mechanical power is required to be very low velocity and high force necessitating a high precision transmission to reduce knee joint speeds and increase knee joint torques (70 rpm peak, and 25 Nm peak, respectively). Relative to piezoelectric material, electroactive polymers accept mechanical power over faster speeds and have a higher efficiency [10]. However, the output electrical power is high voltage and low current necessitating extensive power conversion and consequently decreases in efficiency. Compared with the above methods, a lightweight electromagnetic generator is capable of efficiently converting mechanical power into electrical power in a form suitable for charging a battery. Although the input speed and torque requirements for magnetic generators are not ideal for direct coupling to knee motion, we found them superior to the other alternatives because of the feasibility of designing efficient transmissions to convert the knee joint power into a suitable form. A rotary magnetic generator was superior to a linear magnetic generator because the latter requires long displacements for efficient power generation necessitating a large generator size as well as additional transmission complexity to convert rotary knee motion into a linear form. To increase the angular velocity prior to input into the rotary magnetic generator, we used spur gears because of their high efficiency and relative simplicity. For a more in-depth analyses of biomechanical power conversion methods, we refer the reader to Kysmiss et al. and Niu et al. [32,33].

For a given generator and transmission topology, it is necessary to carefully choose system parameters in order to maximize electrical power generation without adversely affecting the walking motion. For a given knee angular velocity, the characteristics of the transmission, generator and electrical load will collectively contribute to the device reaction torque that will resist knee motion. If the joint power that is normally due to muscles could be replaced entirely by the device, the optimal system parameters would produce a reaction torque equal in shape and magnitude to the joint torque measured during the end of swing extension (Figure 1). However, some of the muscles that are responsible for the swing extension knee joint power simultaneously produce torque about the hip joint. The correct timing and magnitude of this hip joint torque is essential for normal walking. Consequently, it is not desirable to replace all of the muscle-induced knee joint torque with device-induced torque. As an initial approximation of the joint torque that could be replaced with an external device, we chose our system parameters to generate half of the joint torque normally required to walk at our test speed. This equated to 7 Nm of peak torque.

**Figure 2:**
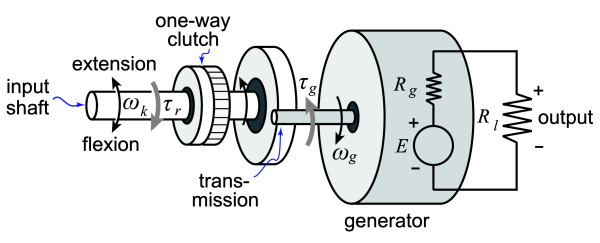
**A simple model of our biomechanical energy harvester**. The input shaft accepts the knee motion at 1:1 ratio through a simple hinge (uni-axis) knee brace. A one-way clutch on the input shaft couples the gear train with knee motion during knee extension, and decouples the gear train from knee motion during knee flexion. The gear train transfers the low speed (*ω*_*k*_) but high torque (*τ*_*r*_) mechanical power into high speed (*ω*_*g*_) and low torque (*τ*_*g*_) mechanical power suitable for power generation. A miniature brushless DC generator converts the mechanical energy in to electrical energy where *E *is the generated electrical potential, *R*_*g *_is the generator terminal resistance and *R*_*l *_is the external electrical load.

There is more than one set of transmission, generator and electrical load parameters that will generate the target reaction torque for a particular knee angular velocity. The optimal set will maximize electrical power generation while minimizing system size and mass. To identify the relevant system parameters and understand their respective contributions to electrical power generation, we considered a simple model for the conversion of knee angular velocity into electrical power by a rotary electromagnetic generator, and the reaction torque resulting from this power generation (Figure 2). In this model, the angular velocity during knee extension, *ω*_*k*_, is first amplified by a gear train before being applied to the generator.

(1)

where *ω*_*g *_is the velocity applied to the generator, and *r*_*t *_is the transmission gear ratio. With the input angular velocity, the generator generates a voltage, *E*:

(2)

where *K*_*g *_is the back electromotive force (EMF) constant. The back-EMF constant is specific to each generator and depends on generator topology and materials. All else being equal, a generator will have a larger *K*_*g *_if it has a greater number of turns or parallel paths in the armature winding, a greater number of poles, or a stronger magnetic flux per pole. When an external electrical load, *R*_*l*_, is connected to the generator, the generated voltage will be divided between the generator's terminal resistance, *R*_*g*_, and the load.

(3)

where *I *is the current in the circuit. Like the back-EMF constant, the terminal resistance depends upon the generator topology and materials. All else being equal, a generator will have a smaller *R*_*g *_if the conducting wires are shorter, have a larger diameter, or have a lower specific resistance. Substituting Equation 1 into Equation 2, and Equation 2 into Equation 3, and then solving for the circuit current yields:

(4)

The efficiency of the generator, ***η***_*g*_, is the ratio of useful electrical power–the power applied to the load–to total electrical power including that dissipated by the generator terminal resistance. This efficiency can be expressed by the following relationship between terminal resistance and electrical load:

(5)

The torque applied by the generator to the gear train, *τ*_*g*_, is a function of the back-EMF constant and the induced current,

(6)

The generator torque is amplified by the gear train before being applied to the knee:

(7)

where *τ*_*r *_is the device reaction torque applied to the knee and ***η***_*t *_is the efficiency of the gear train. Substituting Equation 4 into Equation 6, and Equation 6 into Equation 7, yields the following equation for the torque applied to the knee:

(8)

The system efficiency for converting mechanical power from the knee into electrical power to the load, ***η***, is the product of the transmission efficiency and the generator efficiency:

(9)

These equations illustrate that the transmission gear ratio and transmission efficiency, the generator back-EMF constant and the generator terminal resistance, and the electrical load resistance are the key design parameters for maximizing the system efficiency (Equation 9) while having the device apply the target reaction torque to the knee (Equation 8).

Due to size and mass constraints, the parameters of the device could not be chosen independently. In the generator design, for example, increasing the back-EMF constant by increasing the turns, paths, poles or flux would typically require more material and thus greater size and mass. Similarly, decreasing terminal resistance by using larger diameter conducting wires would result in an increase in material and mass. For a given generator size and mass, there is also an inherent compromise between terminal resistance and back-EMF constant. Increasing the back-EMF constant by increasing the number of turns, parallel paths or poles would require a greater length of conducting wire resulting in a greater terminal resistance assuming the same conducting material is used.

Choosing an optimal set of parameters is complex. For example, an increase in electrical load would increase system efficiency (Equation 9) but not without a reduction in reaction torque (Equation 8). The reduction in reaction torque could be balanced by changing one of the other design parameters, but not without the potential of decreasing system efficiency. Maintaining reaction torque by increasing back-EMF constant with more wire windings or poles, for example, would increase terminal resistance and thus decrease system efficiency (Equation 9). While our algebraic analysis of a simple model illustrates the relevant system parameters as well as their interdependence, it did not allow us to determine the optimal set for our particular energy harvester design because the relationship between these design parameters and the system size and mass is considerably more complicated.

**Figure 3:**
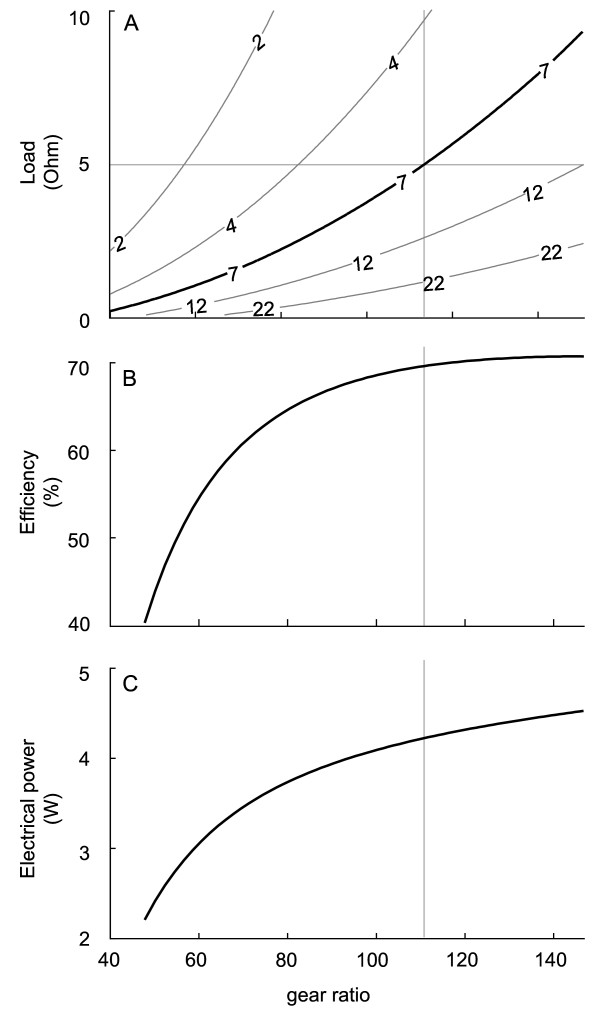
**The simulated device reaction torque, efficiency and generated electrical power depend on the transmission gear ratio and the external electrical load**. A) A contour plot of simulated device reaction torque at different combinations of gear ratio and external load. Each curve is an iso-reaction torque line with the number on the curve illustrating the torque in Nm. The target reaction torque of 7 Nm is illustrated with a thicker line. B) Simulated device mechanical to electrical efficiency at the target reaction torque achieved through different combinations of gear ratio and external load. C) The electrical power generated by the simulated device at the target reaction torque achieved through different combinations of gear ratio and external load. The vertical grey line illustrates the gear ratio used in our current design.

To isolate the generator mass and size from selection of design parameters, we first selected a small, lightweight, efficient and commercially-available rotary magnetic generator (EC45 Flat brushless DC motor; *K*_*g *_= 0.0335*V*/*rad*/*s*; *R*_*g *_= 1.03**Ω**; Mass = 110 g; Maxon Motors, Burlingame, CA). We next determined a combination of gear ratio and electrical load that generated the desired device reaction torque (7 Nm) while maximizing efficiency. For a given angular velocity, the target reaction torque could be achieved with an infinite combination of these two parameters (Figure 3; Equation 8). The efficiency and electrical power output were maximized at the highest gear ratios for a particular reaction torque (Figure 3B and 3C) indicating that we should choose the maximal gear ratio. However, gear diameters could not be made arbitrarily large, due to our size constraint, or arbitrarily small, due to strength requirements. While these constraints could be partially circumvented by increasing the gear ratio through increasing the number of gear train stages, each additional pair of meshing teeth decreases transmission efficiency [34]. We settled on a three-stage design and chose the maximum gear ratio (113:1) that did not exceed our size envelope or make the smallest gears likely to fail. With the selected gear ratio, an electrical load of 5 **Ω **was required to generate the target 7 Nm of peak reaction torque. This choice of parameters predicted 4.2 W of electrical power production at efficiency of 70% (Figure 3).

**Figure 4:**
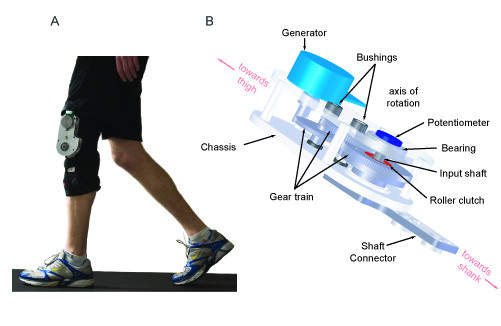
**Biomechanical energy harvester**. A) The device consists of an aluminum chassis and generator mounted on an orthopaedic knee brace. The entire unit weighs about 1.6 kg. While the subject in this image is wearing the device only on his left leg, all human subject testing was conducted with devices worn bilaterally. B) A schematic of the chassis illustrates the location of transmission, generator and sensing components.

We used a customized orthopaedic knee brace to couple the motion of the transmission and generator to the knee motion. Modelling software (SolidWorks, Concord, MA) was used to design an aluminium chassis to house the transmission and generator (Figure 4). The chassis (0.76 kg) was mounted on an orthopaedic knee brace (0.89 kg, GII Trainer; Ossur, Reykjavik) modified to accommodate device components. We choose this particular brace due to its uni-axis knee joint–an uncommon characteristic among knee braces. This selection led to the simplicity of harvester design but had consequences to user comfort because the knee is not a simple hinge joint [35]. We also added thigh and shank extensions to reduce the forces applied to the body by the brace platform. Lower forces not only made the device more comfortable, but also more tightly coupled knee motion to brace hinge motion by reducing soft tissue compression.

The final design challenge was to selectively engage power generation during the end of swing extension. Commercially available mechanical clutches for rapidly coupling the transmission to the input motion are too large, heavy and power hungry. Instead, our device used two mechanisms in series to selectively engage power generation. The first mechanism was mechanical in nature–a passive one-way clutch (S99NH3MURC1616, SDP/SI, New York) was mounted on the first gear and oriented to engage the transmission during knee extension while allowing the input shaft to freely rotate during knee flexion (Figure 4). The second mechanism was electrical–a controllable switch to open and close the power generating circuit. We used a PhotoMOS switch (AQZ202, Panasonic, NJ) to take advantage of its low latency (~2 ms), small on-resistance (0.1 **Ω**) and low power consumption (10 mW).

**Figure 5:**
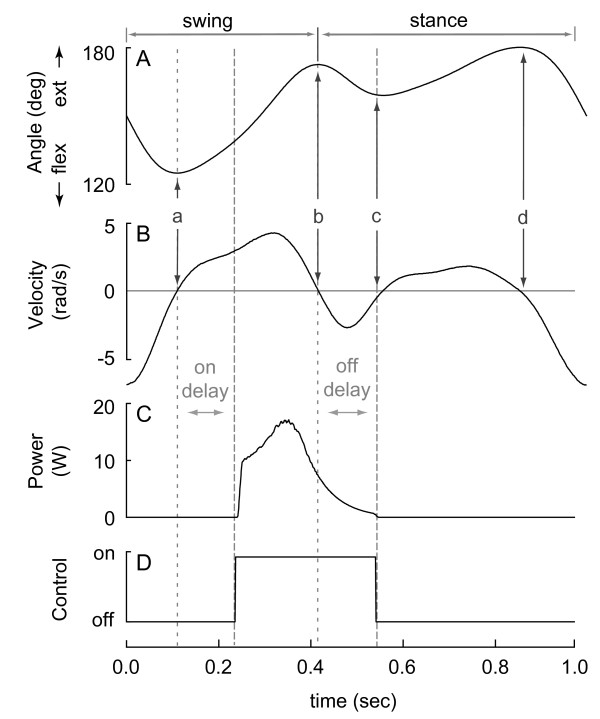
**The control system used knee angle, angular velocity and time delays to engage and disengage power generation**. A) Knee joint angle measured using a potentiometer. B) Knee joint angular velocity determined from time differentiation of the filtered knee joint angle. C) Measured electrical power. D) Control signal with "on" indicating that the switch is closed engaging the power generation circuit. Vertical line a, b, c and d denote the start of swing extension, stance flexion, stance extension and swing flexion, respectively. The control system engages power generation at the end of swing extension by adding a delay to the detected onset of swing extension. The control system disengages power generation before the start of stance extension, but after stance flexion to harvest energy from the inertia of the transmission and generator, by adding a delay to the detected onset of stance flexion.

The control algorithm used knee angle and angular velocity to determine the beginning and end of swing extension (Figure 5). Knee angle was measured from a potentiometer (6639S-1-502, Bourns Inc., CA) mounted on the input shaft (Figure 4). Knee angular velocity was calculated by low-pass filtering knee angle (Second order, Butterworth, 6 Hz cut-off) and then differentiating with respect to time. The control system determined the different phases of the gait cycle by tracking angular velocity zero-crossings, the direction of zero-crossing (upward or downward) and the magnitude of the knee angle at these zero-crossings. Of the two upward zero-crossings, the smaller knee angle indicated the initiation of swing extension (vertical line (a) in Figure 5). The first downward zero crossing after the initiation of swing extension indicated the transition to stance flexion (vertical line (b) in Figure 5).

The control system used time delays to more effectively time the engagement of power generation. Knee flexor muscles become active part of the way into swing extension (Figure 1E). To match this muscle timing, the control system delayed engaging power generation by 70–90 ms from the detected onset of swing extension. Stance flexion follows swing extension, and while the roller clutch prevented the knee from generating additional mechanical power on the transmission during this flexion phase, the transmission and generator were still in motion. Consequently, the control system delayed disengaging power generation by 80 ms from the detected onset of stance flexion in order to harvest the rotational kinetic energy remaining in the transmission and generator while still disengaging in time to avoid power generation from knee extension later in stance. To allow for rapid prototyping, the control system was implemented in Simulink, compiled using Real Time Workshop and executed at 1 kHz using Real Time Windows Target on a desktop computer (Mathworks, Natick, MA). A multifunctional I/O board (NI 6031E, National Instruments Inc, CA) performed the data acquisition of the potentiometer signal and communicated the computer-generated control commands to the switch.

### Device Testing

We operated the device in four different modes. In the *generative braking mode*, the control system selectively engaged and disengaged power generation to target the swing extension negative work region. In the *continuous generation mode*, the control system was deactivated, the power generation circuit was always completed, and electrical power was generated whenever the generator was in motion. In the *flexion dissipation mode*, the control system engaged power generation during the swing and stance knee flexion phases to completely dissipate the kinetic energy in the transmission and generator that had accumulated during knee extension. This testing mode was used to determine the amount of torque and mechanical power produced due to friction and inertia during the knee extension phases, independent of generator back-EMF. In the *disengaged mode*, the roller clutch was manually disengaged so that the transmission was never in motion. This testing mode served as a control condition for human subject experiments to account for any physiological changes that resulted from carrying the added mass independent of physiological changes resulting from energy harvesting.

We used an ergometer to quantify the device reaction torque, mechanical power and efficiency. A commercially available dynamometer, designed to measure knee torque and power under specified kinematic conditions (BIODEX II, Biodex Medical Systems, New York), was modified to include a jig that accepted our device. We also modified its control system to drive the jig with the average knee angles measured during human subject trials while we measured angular velocity, torque and electrical power. The torque required to drive the additional mass of the jig and the brace contributed to the measured torque in the three power generating modes and was responsible for all the measured torque in the disengaged mode. Thus, we calculated the torque applied by the input shaft to the brace (device reaction torque) in the power generating modes by first subtracting the measured torque in the disengaged mode. The mechanical power input into the device was calculated as the product of angular velocity and measured device reaction torque. Efficiency was calculated as the ratio between the average output electrical power and the average input mechanical power over six complete gait cycles.

### Human Subject Testing

The methods of these experiments are presented in detail in our previous publication [15]–we will only briefly summarize them here. We tested the energy harvesting performance on six male subjects walking on a treadmill at 1.5 m·s^-1 ^while wearing a device on each leg. We estimated metabolic cost using a standard respirometry system and measured the electrical power output of the generator. We used the cost of harvesting metric (COH) to make comparisons between different power generating modes. This dimensionless quantity is the additional metabolic power required to generate one Watt of electrical power [15]:

(10)

For conventional generation, we estimated the COH from the efficiency with which the device converts mechanical work to electricity, and the efficiency with which muscles perform positive work:

(11)

## Results and discussion

**Figure 6:**
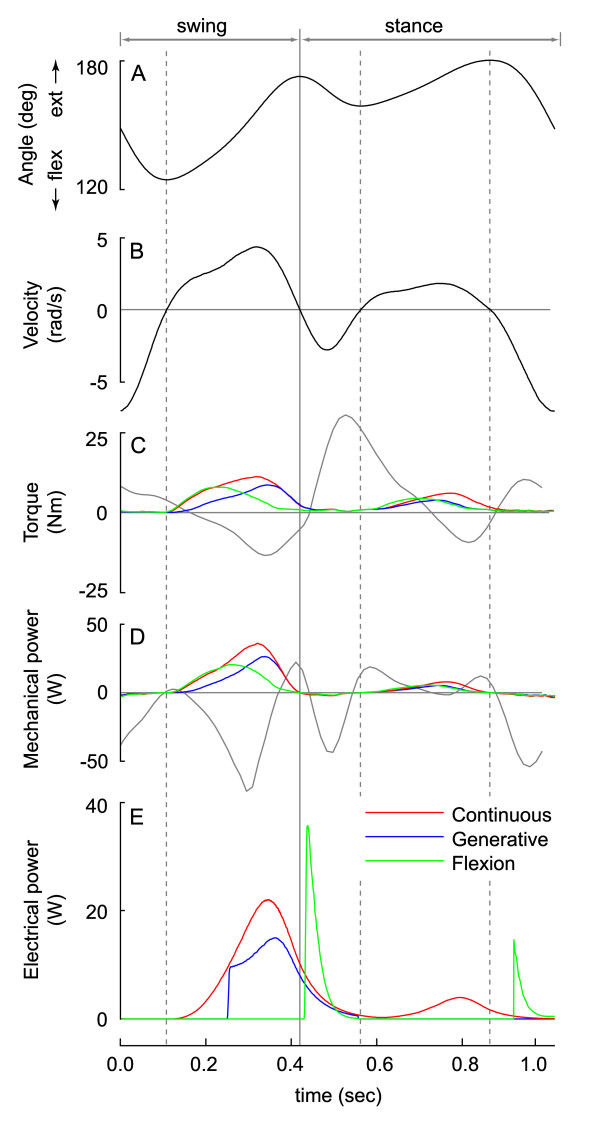
**Test ergometer data from one simulated walking stride cycle**. A) Joint angle. B) Joint angular velocity. Measured device reaction torque (C), mechanical power (D), and electrical power (E) during continuous generation, generative braking, and flexion dissipation modes. The grey lines in C and D indicate typical knee joint torque and power during walking at a similar speed. Regions corresponding approximately to swing and stance phases are indicated for reference.

During the ergometer testing of the flexion dissipation mode, the measured device reaction torque and mechanical power during swing and stance extension phases were purely due to the inertial and frictional forces required to drive the transmission and generator. The peak device reaction torque (5.9 Nm) and peak input mechanical power (20.6 W) occurred during swing extension when the acceleration-dependent inertial torque was the highest (Figure 6). Due to a lower angular velocity, peak stance extension torque and peak mechanical power were both smaller than in swing extension (3.3 Nm and 5.5 W, respectively). The average mechanical power for a complete cycle was 4.4 W.

By completing the power generating circuit for the whole stride cycle, the continuous generation mode produced greater peak torque, peak input mechanical power and average mechanical power when compared with the flexion dissipation mode. The peak device torque (8.4 Nm) and peak input mechanical power (36.0 W) occurred later in swing extension and represented a 42% and 75% increase over the flexion dissipation mode, respectively. The increases were purely due to the generator back-EMF. Because of the slower angular velocity, the stance extension peak torque (4.5 Nm) and peak input mechanical power (7.9 W) were only 54% and 22% of the swing extension values, respectively. The average input mechanical power was 6.8 W, a 55% increase over the flexion dissipation mode. The output electrical power was 4.4 W resulting in an efficiency of 64.7%. To determine the sensitivity of the calculated efficiency to the variation of knee kinematics, we scaled the input angular velocity profile by ± 10% and found only small changes in the efficiency (< 3%).

In the generative braking mode, selectively engaging and disengaging the power generating circuit effectively controlled the magnitude and timing of the resulting device reaction torque applied to a user. Torque increased slowly in the beginning of swing extension and reached peak torque (6.4 Nm) towards the end of swing (Figure 6C), matching well the timing of normal knee joint torque and knee flexor activity (Figure 1). The lower torque during early swing extension, compared with continuous generation, substantially reduced the resistance to knee extension at a phase in the gait cycle when this motion is normally nearly passive [29,36]. The lower torque was partially due to flywheel-like behaviour of the transmission and generator–they remained in motion during early swing extension from their initial acceleration during stance extension. At peak torque, the contribution from inertia and friction was only 2.0 Nm, determined from the flexion dissipation mode. Thus the generator EMF was responsible for 70% of the peak reaction torque, indicating that closing the power generation circuit was an effective method for controlling mechanical resistance to knee motion. Compared with continuous generation, stance extension peak torque (2.9 Nm) and peak input mechanical power (5.3 W) were reduced by 36% and 33%, respectively. Torque was still required to overcome friction and inertia in the transmission and generator though the power generating circuit was open. The average input mechanical power and output electrical power were 4.4 W mechanical and 2.4 W electrical resulting in an efficiency of 54.6%. The efficiency in generative braking mode was lower than in continuous generation because the device spent a greater amount of time dissipating mechanical energy without producing electrical power.

Using our device, the cost of harvesting by conventional generation would be approximately 6.2–each additional Watt of electricity would require 6.2 Watts of metabolic power. This was estimated from the peak device efficiency (64.7%) and the peak efficiency of performing positive muscle work (25%) (Equation 11). The COH in generative braking (0.7 ± 4.4)–calculated from dividing the additional metabolic power required for generative braking relative to that required for the disengaged mode (5 ± 21 W) by the measured electrical power (4.8 ± 0.8 W)–was substantially lower than for conventional generation indicating that it did not depend entirely upon additional positive muscle work to produce electricity. That we measured a slight increase in metabolic cost indicated that generative braking did not simply replace negative muscle work. If it had done so, we would have expected a 7.3 W decrease in metabolic cost calculated by dividing the measured electrical power by the product of the device efficiency (54.6%) and the efficiency of performing negative muscle work (-120%). The likely source for the additional metabolic cost is the positive muscle work required to overcome the added resistance during stance extension (Figure 6). The continuous generation COH (2.3 ± 3.0) fell between that for generative braking and that for conventional generation indicating that its electrical power production (7.0 ± 0.7 W) was partially by conventional generation with a high COH and partially by generative braking with a very low COH.

While generating power was economical, walking while wearing the device was not. In our previous human experiments [15], we had included a normal walking condition in which subjects walked on the treadmill without wearing the device and found that the disengaged mode required an average metabolic power of 366 ± 63 W compared to 307 ± 64 W for walking without wearing the device, a 19.2% increase (p = 1.1e-5). This increase in metabolic cost was due entirely to carrying the additional mass as the device did not resist knee motion in the disengaged mode.

## Conclusion

We have developed a biomechanical energy harvester for generating electricity from walking. The device operated about the knee to take advantage of the large amount of negative work that muscles perform about this joint. It used a one-way clutch to transmit only knee extensor motions, a spur gear transmission to amplify the angular speed, a brushless DC rotary magnetic generator to convert the mechanical power into electrical power, and a control system to determine when to engage and disengage the power generation based on measurements of knee angle. A customized orthopaedic knee brace supported the hardware and distributed the device reaction torque over a large leg surface area. For convenient experimentation, the control system resided on a desktop computer and resistors dissipated the generated electrical power. The device was efficient and the control system was effective at selectively engaging power generation. Consequently, subjects were able to generate substantial amounts of electrical power with little additional effort over that required to support the device mass.

To prove useful in practical implementations, the metabolic cost of carrying the device will have to be decreased. Revisions to improve the fit, weight, and efficiency of the device can not only reduce the cost, but can also increase the generated electricity. In particular, next generation devices would benefit from a more form-fitting knee brace made out of lighter weight material such as carbon fibre. A generator designed specifically for this application could have lower internal losses and inertia while requiring a smaller and lighter gear train. Because the metabolic cost of carrying a given mass proximally is considerably cheaper than carrying it distally [37], the largest reduction in the cost of carrying the device will likely come from relocating the components higher on the thigh.

While we have focused on harvesting energy from swing extension, power generation is possible from other periods of the gait cycle. At the beginning of the stance phase, for example, the knee flexes while the knee extensor muscles generate an extensor torque performing substantial negative work to aid in the redirection of the centre of mass velocity (Figure 1) [38]. The amount of available energy at moderate walking speeds is only slightly less than that at the end of swing and it increases strongly with speed [30]. Consequently, our initial device design attempted to also harvest energy from stance flexion. It used two oppositely-oriented roller clutches on the input shaft, causing the generator to spin in the same direction regardless of the direction of knee motion, and an extra stage of gearing to increase the gear ratio during flexion. While the higher gear ratio was required to better match the low angular velocity and high torque characteristics of stance flexion mechanical power (Figure 1), the transmission and generator friction and inertia presented awkwardly large resistive forces during the high angular velocity swing flexion phase. This was not an issue for knee extension where power generation was engaged during swing extension, when knee angular velocity is high, and disengaged during stance extension, when knee angular velocity is low. While this drawback forced us to disregard power generation during stance flexion, power generation could be doubled with a more suitable design. For now, generative braking during stance flexion is best considered a hypothesis that must be tested empirically as it is not yet known how much of the negative work during this period is stored and subsequently returned during stance extension.

While future versions of this technology may prove useful to the general public for powering their portable devices, people whose lives depend on portable power will embrace it more quickly. Energy harvesting to trickle charge batteries in current computerized and motorized prosthetic limbs, for example, would allow amputees to walk further and faster [39-41]. It would also enable future powered prosthetic and orthotic technologies to become more sophisticated by alleviating some of the limitations that batteries currently place on their design. The key principles are considerably more general than the current embodiment–they extend to joints other than the knee and to movements other than walking. The principles could also be embodied in a fully implanted energy harvester to power neurostimulators, drug pumps and other implantable devices. Irrespective of if they are embodied in a wearable or implanted design, energy harvesters that operate about body joints and selectively engage power generation have the potential to improve the quality of life for the user without increasing their effort.

## Competing interests

QL, VN and JMD have equity interest in Bionic Power Incorporated, a company that performs research and development on the energy harvesting technology reported in this paper. JMD is chief science officer and board member of Bionic Power Incorporated.

## Authors' contributions

QL took the lead role in device design and analysis, experiment design and analysis, and manuscript writing. VN took the lead role in designing the figures and supported QL in performing the experiments and analyzing the results. JMD supported QL and VN in harvester design and analysis, experiment design and analysis, figure design and worked closely with QL in writing the manuscript. All authors read and approved the final manuscript.

## References

[B1] Starner T, Paradiso JA, Piguet C (2005). Human generated power for mobile electronics. Low-power electronics design.

[B2] Nokia 6301 data sheet. http://www.nokia.com/A4140021.

[B3] Dell™ Inspiron™ 1525/1526 product information. http://support.dell.com/support/edocs/systems/ins1525/en/index.htm.

[B4] Ottobock C Leg. http://www.ottobock.com/cps/rde/xbcr/ob_com_en/ifu_647h215_d_gb_f_e_c_leg.pdf.

[B5] Ossur Proprio foot technical manual. http://www.ossur.com/lisalib/getfile.aspx?itemid=12360.

[B6] Ossur Rheo knee technical manual. http://www.ossur.com/lisalib/getfile.aspx?itemid=7000.

[B7] Margaria R (1968). Positive and negative work performances and their efficiencies in human locomotion. Int Z Angew Physiol.

[B8] Brooks GA, Fahey TD, Baldwin KM (2005). Exercise physiology: human bioenergetics and its applications.

[B9] Starner T (1996). Human-powered wearable computing. IBM Systems Journal.

[B10] Paradiso JA, Starner T (2005). Energy scavenging for mobile and wireless electronics. IEEE Pervasive Computing.

[B11] Chapuis A, Jaquet E (1956). The History of the self-winding watch, 1770–1931.

[B12] Rome LC (2004). Backpack for harvesting electrical energy during walking and for minimizing shoulder strain.

[B13] Rome LC, Flynn L, Goldman EM, Yoo TD (2005). Generating electricity while walking with loads. Science.

[B14] Kornbluh RD, Pelrine RE, Pei Q, Heydt R, Stanford SE, Oh S, Eckerle J (2002). Electroelastomers: Applications of dielectric elastomer transducers for actuation, generation and smart structures. Proceedings of the SPIE – Smart Structures and Materials 2002: Industrial and Commercial Applications of Smart Structures Technologies.

[B15] Donelan JM, Li Q, Naing V, Hoffer JA, Weber DJ, Kuo AD (2008). Biomechanical energy harvesting: generating electricity during walking with minimal user effort. Science.

[B16] van Ingen Schenau GJ, Cavanagh PR (1990). Power equations in endurance sports. J Biomech.

[B17] Demirdoven N, Deutch J (2004). Hybrid cars now, fuel cell cars later. Science.

[B18] Pugh LG (1971). The influence of wind resistance in running and walking and the mechanical efficiency of work against horizontal or vertical forces. J Physiol.

[B19] Webb P, Saris WHM, Schoffelen PFM, Schenau GJV, Tenhoor F (1988). The Work of Walking – a Calorimetric Study. Med Sci Sports Exerc.

[B20] DeVita P, Helseth J, Hortobagyi T (2007). Muscles do more positive than negative work in human locomotion. J Exp Biol.

[B21] Donelan JM, Kram R, Kuo AD (2002). Simultaneous positive and negative external mechanical work in human walking. J Biomech.

[B22] Donelan JM, Kram R, Kuo AD (2001). Mechanical and metabolic determinants of the preferred step width in human walking. Proc Biol Sci.

[B23] Donelan JM, Kram R, Kuo AD (2002). Mechanical work for step-to-step transitions is a major determinant of the metabolic cost of human walking. J Exp Biol.

[B24] Winter DA (1990). Biomechanics and motor control of human movement.

[B25] Hof AL, Elzinga H, Grimmius W, Halbertsma JP (2002). Speed dependence of averaged EMG profiles in walking. Gait & Posture.

[B26] Eng JJ, Winter DA (1995). Kinetic analysis of the lower limbs during walking: what information can be gained from a three-dimensional model?. J Biomech.

[B27] Zajac FE (1993). Muscle coordination of movement: a perspective. J Biomech.

[B28] Alexander RM (1990). Three Uses for Springs in Legged Locomotion. International Journal of Robotics Research.

[B29] McMahon TA (1984). Muscles, reflexes, and locomotion.

[B30] Winter DA (1983). Energy generation and absorption at the ankle and knee during fast, natural, and slow cadences. Clin Orthop.

[B31] Delp SL, Arnold AS, Speers RA, Moore CA (1996). Hamstrings and psoas lengths during normal and crouch gait: Implications for muscle-tendon surgery. J Orthop Res.

[B32] Kymissis J, Kendall C, Paradiso JA, Gershenfeld N (1998). Parasitic Power Harvesting in Shoes. Second IEEE International Conference on Wearable Computing; Oct.

[B33] Niu P, Chapman P, Riemer R, Zhang X (2004). Evaluation of Motions and Actuation Methods for Biomechanical Energy Harvesting. 35th Annual IEEE Power Electronics Specialists Conference; Aachen Germany IEEE.

[B34] Buckingham E (1928). Spur gears design, operation, and production.

[B35] Kurosawa H, Walker PS, Abe S, Garg A, Hunter T (1985). Geometry and motion of the knee for implant and orthotic design. J Biomech.

[B36] Mochon S, McMahon TA (1980). Ballistic walking. J Biomech.

[B37] Soule RG, Goldman RF (1969). Energy cost of loads carried on the head, hands, or feet. J Appl Physiol.

[B38] Kuo AD, Donelan JM, Ruina A (2005). Energetic consequences of walking like an inverted pendulum: step-to-step transitions. Exerc Sport Sci Rev.

[B39] Berry D (2006). Microprocessor prosthetic knees. Phys Med Rehabil Clin N Am.

[B40] Johansson JL, Sherrill DM, Riley PO, Bonato P, Herr H (2005). A clinical comparison of variable-damping and mechanically passive prosthetic knee devices. Am J Phys Med Rehabil.

[B41] Seymour R, Engbretson B, Kott K, Ordway N, Brooks G, Crannell J, Hickernell E, Wheeler K (2007). Comparison between the C-leg microprocessor-controlled prosthetic knee and non-microprocessor control prosthetic knees: a preliminary study of energy expenditure, obstacle course performance, and quality of life survey. Prosthet Orthot Int.

